# Laryngeal ultrasound for the prediction of severe laryngomalacia

**DOI:** 10.1371/journal.pone.0326439

**Published:** 2025-06-25

**Authors:** Arisa Duantaweesook, Vannipa Vathanophas, Kitirat Ungkanont, Archwin Tanphaichitr, Thanakrit Wannarong, Ramida Amornsitthiwat

**Affiliations:** 1 Department of Otolaryngology, Faculty of Medicine, Naresuan University, Phitsanulok, Thailand; 2 Department of Otorhinolaryngology, Faculty of Medicine Siriraj Hospital, Mahidol University, Bangkok, Thailand; 3 Department of Radiology, Faculty of Medicine Siriraj Hospital, Mahidol University, Bangkok, Thailand; Universiti Malaya Fakulti Perubatan: University of Malaya Faculty of Medicine, MALAYSIA

## Abstract

**Background:**

Laryngeal ultrasound (LUS) is a noninvasive, painless, and radiation-free imaging method that presents a promising alternative, especially for the dynamic assessment of laryngeal structures. It can also be utilized by general practitioners. This study assesses the diagnostic accuracy, patient comfort, and cost-effectiveness of LUS compared to flexible fiberoptic laryngoscopy (FFL) for diagnosing laryngomalacia in infants aged ≤ 2 years with stridor-specific airway issues.

**Methods:**

A total of 43 infants presented with inspiratory stridor or other airway symptoms and underwent assessments using both flexible fiberoptic laryngoscopy and laryngeal ultrasound. Laryngomalacia was diagnosed based on a vocal fold–arytenoid abduction angle of ≤120° and arytenoid and/or vocal fold collapse during inspiration. The diagnostic performance of LUS was compared with FFL, and the severity of the disease was evaluated. Both the pediatric endoscopist and radiologist were blinded to the patients’ diagnoses and study results.

**Results:**

A total of participants was included in the study, comprising 35% males and 65% females. The mean age of 4.52 ± 5.44 months and the mean weight of 4.62 ± 2.23 kg. Laryngomalacia was diagnosed in 60% of cases using FFL, with seven infants requiring surgical intervention; all were also identified as having laryngomalacia via LUS. LUS demonstrated diagnostic efficiency for laryngomalacia in 7 out of 19 infants, with a sensitivity of 26.92%, specificity of 100%, negative predictive value of 47.22%, and positive predictive value of 100%. The overall diagnostic accuracy of LUS was 55.8%.

**Conclusion:**

While LUS cannot replace FFL as the primary diagnostic tool for infant laryngomalacia, it serves as a valuable adjunct for follow-up assessments and for identifying moderate to severe cases that may require surgical intervention. Further research and advancements in ultrasound technology may enhance diagnostic accuracy and broaden clinical applications for general practitioners.

## Background

Laryngomalacia is the predominant cause of stridor and upper airway obstruction in infants under 18 months, accounting for 45–75% of laryngeal disorders, with approximately 10% of cases being severe [1–3]. This condition is characterized by the collapse of supraglottic structures, such as the epiglottis and aryepiglottic folds, during inspiration. Key symptoms include intermittent, low-pitched inspiratory stridor that worsens during activities like crying or feeding. While most cases are resolved by 12–18 months, severe instances can lead to complications such as feeding difficulties, failure to thrive, or cardiac issues. Around 10% of affected infants require surgical intervention.

Flexible fiberoptic laryngoscopy is the gold standard for diagnosing laryngomalacia in infants, as it enables the identification of dynamic supraglottic narrowing and prolapse of airway structures. However, FFL has notable drawbacks, including the risk of laryngospasm, patient discomfort, and high costs [4]. Moreover, imaging modalities such as CT, MRI, and fluoroscopy are less effective in diagnosing laryngomalacia due to their inability to capture dynamic movement and the need for sedation or anesthesia in infants [5]. This necessitates a simpler, noninvasive, and effective diagnostic alternative.

Laryngeal ultrasound has gained attention as a noninvasive and radiation-free imaging modality for assessing laryngeal structures. While transcutaneous laryngeal ultrasonography (TLUS) is well-documented for evaluating vocal fold mobility and diagnosing vocal cord paralysis in adults [6], this study reviews its effectiveness in assessing vocal cord function based on cases at a tertiary government hospital. It also explored its application in pediatric populations, particularly in diagnosing conditions such as laryngomalacia and subglottic stenosis. Garel et al. demonstrated the ability of LUS to visualize normal laryngeal anatomy and dynamic movement in infants, establishing its diagnostic potential [7]. Similarly, Ongkasuwan et al. reported that LUS is comparable to FFL in assessing vocal fold mobility in critically ill children [8]. Despite this growing body of evidence, the use of LUS specifically for diagnosing laryngomalacia across varying severities remains underexplored.

By incorporating dynamic assessments of the vocal fold–arytenoid angle and arytenoid collapse, this study aims to evaluate the diagnostic efficacy of LUS compared to FFL, focusing on its utility in detecting moderate to severe laryngomalacia in infants under two years old airway symptoms according to a combination of diagnostic criteria.

### Objective

This study aimed to evaluate the diagnostic performance of LUS compared to FFL in diagnosing laryngomalacia and predicting its severity in infants aged ≤ 2 years who present with stridor.

## Materials and methods

### Study design

This was a diagnostic study in infants in the Department of Otorhinolaryngology and Department of Pediatrics, Faculty of Medicine Siriraj Hospital, Mahidol University, Bangkok, Thailand, from July 2021 to January 2023. The study was approved by the Siriraj Institutional Review Board (SIRB), COA no. Si 402/2021; approval date: June 1, 2021

### Subjects

Inclusion criteria encompassed infants aged ≤ 2 years with stridor, treated at the Department of Otorhinolaryngology and Department of Pediatrics, Faculty of Medicine, Siriraj Hospital, Mahidol University, Bangkok, Thailand. All eligible patients underwent assessments using both FFL and LUS. Exclusion criteria included infants with a history of endotracheal intubation, tracheostomy, prior laryngeal surgery, an incongruent examination interval of more than one month between LUS and FFL, failure to complete follow-up, or lack of parental consent.

### Sample size calculation

The sample size was calculated based on data from a previous study by Huilian Huang et al. [9], the study examined the role of laryngeal ultrasound in diagnosing infant laryngomalacia, reporting a sensitivity of 96.3%, specificity of 84.6%. Based on an overall accuracy of 88% and a margin of error of 10%, the required sample size for the present study was 43 participants.

### Methods

The study details were explained to the parents or guardians, and written informed consent was obtained. Patients underwent Laryngeal Ultrasound and Flexible Fiberoptic Laryngoscopy, performed by a pediatric radiologist and ENT specialists, both blinded to diagnosis and results. FFL, using a 2.1 mm scope, was conducted in the operating room to assess inspiratory stridor and laryngeal symptoms. LUS was performed using a GE LOGIQ S8 (GE Healthcare, USA) and a Toshiba Xario 100 (Toshiba, Japan) and a 6–15 MHz linear high-frequency probe in “small parts” mode. During the procedure, each patient, without sedation, remained in a supine position with slight neck extension, while a nurse stabilized the head. Transverse ultrasound scans of the anterior neck were conducted during both crying and quiet respiration. The recorded images and videos ranged from the hyoid to cricoid levels. Notably, longitudinal scans were not performed due to the large probe size, which impeded clear image acquisition in infants. The procedure was conducted on both inpatients and outpatients without causing any pain, and the recorded images included the epiglottis, aryepiglottic folds, and vocal cords during respiration.

To ensure consistency in examination intervals, LUS and FFL were performed within one month of each other. Despite nine patients not completing the LUS follow-up, all available data were documented in report forms for analysis.

### Data collection

Before initiating the study, informed consent was obtained from the parents or legal guardians of all participants. A comprehensive explanation of the study’s objectives, procedures, potential risks, and benefits was provided to ensure complete understanding. Following consent, demographic and clinical data; including age, sex, weight, and underlying medical conditions, were collected.

Flexible Fiberoptic Laryngoscopy was performed using standardized techniques by a physician blinded to the LUS results. Laryngomalacia was classified based on the Olney classification [3] into three distinct types: (1) prolapse of the mucosa overlying the arytenoid cartilages, (2) foreshortened aryepiglottic folds, and (3) posterior displacement of the epiglottis.

**Fig 1:**
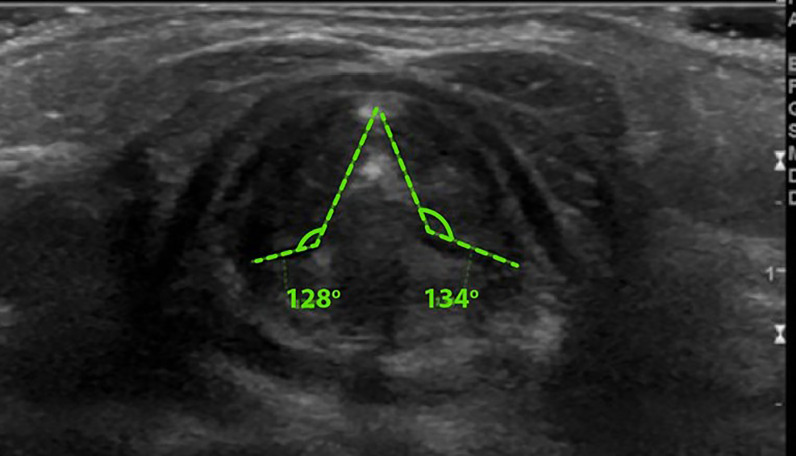
Vocal Fold – Arytenoid angle in abduction > 120 degree = Normal^6.^

**Fig 2:**
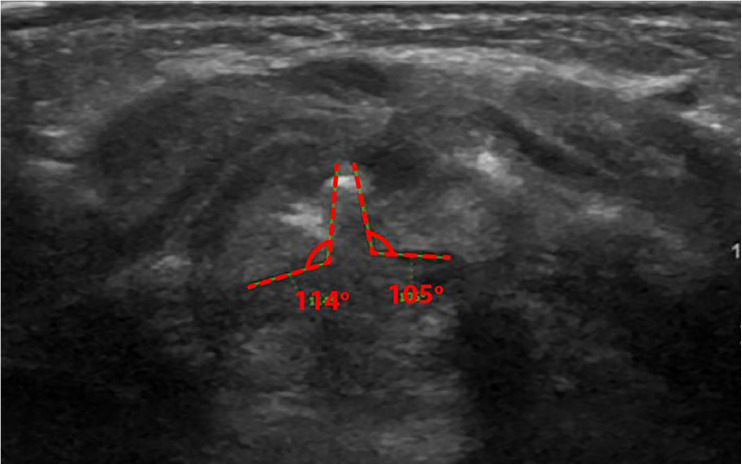
Vocal fold-arytenoid angle is less than and equal to 120 degree and Arytenoid collapse.

The diagnostic interpretation used both static and dynamic image recordings. Static images measured the vocal fold–arytenoid angle, while dynamic assessments evaluated arytenoid adduction and/or vocal fold collapse. A vocal fold–arytenoid angle greater than 120 degrees during abduction indicated normal vocal fold movement [8,10] (Fig 1). Conversely, an angle of 120 degrees or less, coupled with arytenoid adduction and/or vocal fold collapse during inspiration, was indicative of laryngomalacia [8,9,11,12] (Fig 2). Furthermore, the presence of an omega-shaped epiglottis served as an additional diagnostic feature for laryngomalacia [7]. All interpretations of FFL and LUS were meticulously documented for further analysis.

### Statistical analysis

Descriptive statistics were used to summarize the data. The diagnostic performance of LUS compared to FFL was presented as sensitivity, specificity, accuracy, positive and negative predictive values (PPV, NPV). Statistical analysis was conducted using 2BM SPSS 29.0.

## Results

A total of 52 patients meeting the inclusion criteria were enrolled in this study; however, 9 were lost to follow-up for the ultrasound examination. The final analysis included 43 children with inspiratory stridor (35% boys and 65% girls), with a mean age of 4.52 ± 5.44 months (range, 3 days to 665 days) and a mean weight of 4.62 ± 2.23 kg.

Flexible Fiberoptic Laryngoscopy diagnosed laryngomalacia in 26 out of 43 patients (60%) and non-laryngomalacia conditions in 17 patients (40%). The gender distribution was equal among those diagnosed with laryngomalacia.

Laryngeal Ultrasound identified laryngomalacia in 7 patients (16.3%) and non-laryngomalacia in 36 patients (83.7%). Of the 26 infants diagnosed with laryngomalacia by FFL, LUS accurately detected the condition in 7 infants (27%) in Table 1. LUS failed to diagnose laryngomalacia in 19 infants, 8 of whom were entirely missed, and 11 had mild cases. None of the missed cases required surgery, while those identified by LUS suggested the need for surgical intervention.

**Table 1:** Contingency table and diagnostic performance metrics for Laryngeal Ultrasound (LUS) and Flexible Fiberoptic Laryngoscopy (FFL).

LUS Status	FFL (+)	FFL (-)	Total	Metrics	Value (%)	95% Confidence Interval (CI)
LUS (+)	7 (26.9%)	0 (0.0%)	7 (100%)	Sensitivity	26.9	(11.6, 47.8)
LUS (-)	19 (73.1%)	17 (100%)	36 (100%)	Specificity	100.0	(80.5, 100.0)
Total	26 (100%)	17 (100%)	43 (100%)	Accuracy	55.8	(39.9, 70.9)

LUS showed 100% concordance with FFL in ruling out laryngomalacia in 17 infants. All 7 infants diagnosed with laryngomalacia by LUS were confirmed by FFL. Of the 36 infants classified as non-laryngomalacia by LUS, 17 were also confirmed by FFL.

Overall, LUS demonstrated a sensitivity of 26.92%, specificity of 100%, positive predictive value of 100%, negative predictive value of 47.22%, and an accuracy of 55.8%, using FFL as the reference standard in Table 1. None of the infants diagnosed with laryngomalacia by both LUS and FFL showed any additional airway pathologies, and no false-positive diagnoses were made by LUS utilization.

## Discussion

Our study introduced the approach of using LUS for prediction of severe laryngomalacia in infants. Results showed its specificity and reliability by 100% for distinguishing moderate to severe cases from mild cases even though its sensitivity is limited to 26.92% when compared to FFL. This study demonstrated that while LUS has limited sensitivity (26.92%) compared to FFL, its specificity and positive predictive value of 100% underscores its reliability in ruling out laryngomalacia and identifying moderate to severe cases that may require surgical intervention.

Among the 43 children presented with inspiratory stridor, 60% of laryngomalacia could be identified with FFL, while only 16.3% were diagnosed with LUS. Compared to FFL, LUS demonstrated a sensitivity of 26.92% and a specificity of 100%. While LUS effectively detected severe cases, it missed milder ones. Both methods were fully concordant in identifying non-laryngomalacia cases, and no patients required surgery based on the LUS diagnosis.

In our study, LUS showed lower sensitivity compared to other reports, likely due to our use of two diagnostic criteria: (1) vocal fold-arytenoid angle and (2) vocal fold collapse during inspiration, whereas other studies used either criterion independently. Additionally, while the omega-shaped epiglottis was helpful for differential diagnosis, it was not a definitive criterion for laryngomalacia. Among the 19 infants in whom LUS missed the diagnosis, all had very mild laryngomalacia and did not require surgery, suggesting that LUS may be less sensitive in detecting milder cases of laryngomalacia.

Conversely, LUS correctly identified moderate to severe laryngomalacia in 7 infants, all of whom required surgical intervention. Therefore, when LUS detects laryngomalacia using our criteria, it is likely that surgical intervention will be necessary.

LUS is a noninvasive and radiation-free imaging tool that may serve as an adjunct to FFL in assessing laryngomalacia, particularly in identifying moderate to severe cases that may require surgical intervention. However, given its limited sensitivity and overall accuracy, LUS cannot replace FFL as the primary diagnostic tool for laryngomalacia. Rather, its role may be more relevant in specific clinical scenarios, such as follow-up evaluations or in settings where FFL is unavailable or contraindicated. While LUS showed a high specificity and positive predictive value, it was less effective in detecting milder cases, suggesting that its utility may be more pronounced in those who present with significant airway compromise.

This study has several limitations. First, it was a single-center study with a small sample size. However, we focused on infants up to 2 years old with airway problems—a population that has been underreported in the literature. Future studies should include larger sample sizes and multicenter participation to enhance generalizability. Second, LUS was performed by a single radiologist to minimize inter-observer variability, which could have affected the results. Ensuring ultrasound quality requires skilled operators who can perform examinations consistently; in our study, the operator was blinded to the FFL results to eliminate potential bias. Lastly, LUS is limited in that it cannot detect anomalies in the trachea and bronchi, restricting its diagnostic scope. Further studies with clearly defined patient subgroups utilizing pre-diagnostic criteria are highly suggested for better characterization of the settings in which LUS can be the most beneficial.

## Conclusion

Utilizing laryngeal ultrasound to assess the vocal fold–arytenoid angle in abduction (≤120°) and to detect arytenoid adduction or vocal fold collapse during inspiration can aid in diagnosing infant laryngomalacia, complementing the gold standard flexible fiberoptic laryngoscopy. While LUS cannot replace FFL as the primary diagnostic tool for infant laryngomalacia, it serves as a valuable adjunct for follow-up assessments and identifying moderate to severe cases that may require surgical intervention. However, LUS is a promising imaging modality that complements traditional methods and offers a safer, less invasive option for assessing laryngeal conditions in infants for general practitioners. Further research and advancements in ultrasound technology may enhance diagnostic accuracy and broaden clinical applications.
